# Pathological Impact of the Interaction of NO and CO with Mitochondria in Critical Care Diseases

**DOI:** 10.3389/fmed.2017.00223

**Published:** 2017-12-22

**Authors:** J. Catharina Duvigneau, Andrey V. Kozlov

**Affiliations:** ^1^Institute of Medical Biochemistry, Department of Biomedical Sciences, University of Veterinary Medicine, Vienna, Austria; ^2^Ludwig Boltzmann Institute for Experimental and Clinical Traumatology, Vienna, Austria

**Keywords:** heme oxygenase, nitric oxide synthase, mitochondria, intensive care medicine, carbon monoxide

## Abstract

The outcome of patients with critical care diseases (CCD) such as sepsis, hemorrhagic shock, or trauma is often associated with mitochondrial dysfunction. In turn, mitochondrial dysfunction is frequently induced upon interaction with nitric oxide (NO) and carbon monoxide (CO), two gaseous messengers formed in the body by NO synthase (NOS) and heme oxygenase (HO), respectively. Both, NOS and HO are upregulated in the majority of CCD. A multitude of factors that are associated with the pathology of CCD exert a potential to interfere with mitochondrial function or the effects of the gaseous messengers. From these, four major factors can be identified that directly influence the effects of NO and CO on mitochondria and which are defined by (i) local concentration of NO and/or CO, (ii) tissue oxygenation, (iii) redox status of cells in terms of facilitating or inhibiting reactive oxygen species formation, and (iv) the degree of tissue acidosis. The combination of these four factors in specific pathological situations defines whether effects of NO and CO are beneficial or deleterious.

## Introduction

The majority of patients admitted to intensive care units are suffering from sepsis, septic shock, traumatic hemorrhagic shock (HTS), and traumatic brain injury (TBI). There is plenty of literature suggesting that gaseous messengers, especially NO and CO, play a key role in those life threatening pathological situations (see Table 1). In critical care diseases (CCD), two major pathological processes are operating, namely, impaired tissue perfusion causing tissue hypoxia and induction of an inflammatory host response either in a septic or in an aseptic way. Both processes have been shown to induce NO and CO generation (1, 2). The analysis of literature, in terms of the number of publications, shows the most strong association between NO and sepsis. Also, CO generation is associated mainly with sepsis. For HTS and TBI, the number of publications is much lower. The number of papers showing an association of CCD with NO is more than 10 times higher compared to those with CO (Table 1). A similar trend is found for the association of these gaseous messengers in combination with mitochondria, when the publications are stratified for CCD (Table 2).

**Table 1:** The number of publications in PubMed (until 09-2017) providing evidence for a critical role of NO and CO in critical care diseases [sepsis, traumatic hemorrhagic shock (HTS), and traumatic brain injury (TBI)].

Topic	NO	CO
Sepsis	3765	198
HTS	428	51
TBI	353	29

**Table 2:** The number of publications in PubMed (until 09-2017) providing evidence for a critical role of NO and CO in combination with mitochondria in critical care diseases [sepsis, traumatic hemorrhagic shock (HTS), and traumatic brain injury (TBI)].

Topic	NO	CO
Sepsis + mitos	112	13
HTS + mitos	9	4
TBI + mitos	17	0

Although analyses of mitochondrial function in patients with CCD are limited due to the restricted availability of samples, the results of these studies point towards a critical contribution of mitochondrial dysfunction to deleterious outcome (3–12).

In patients suffering from septic shock, mitochondrial dysfunction was most pronounced in non-survivors (4, 10). It was also suggested that not only mitochondrial dysfunction but also a decreased mitochondrial content may compromise bioenergetics status of respective tissues (6, 7). However, mitochondrial function/dysfunction appears to vary substantially among subjects suffering from CCD, organs are affected to a different degree, and mitochondrial function/dysfunction may change during disease progression (13). Therefore, the relevance of mitochondrial dysfunction as denominator of CCD outcome is avidly discussed.

The aim of this review is to highlight the mechanisms that are associated with the modulation of mitochondrial function mediated by NO and CO, which may critically influence the outcome of CCD patients. Meanwhile, the mechanisms of NO and CO mediated regulation of mitochondrial function are relatively well understood. However, in CCD a multitude of factors combine which all have the potential to interfere with mitochondrial function or with the effects of gaseous messengers. To draw conclusions about this interaction basing on literature it is necessary to have an overview of how the data on mitochondrial function, NO and CO levels were obtained, and which metabolic steps of NO and CO synthesis and elimination have to be considered.

### Role of Inflammatory Response in CCD for NO and CO

The septic inflammatory response is induced by pathogens entering the body after infectious diseases or traumatic injury. Pathogens or their toxins activate the innate immune cells *via* danger signals derived from pathogens (PAMPs) and lead to an inflammatory host response, a phased reaction including systemic inflammatory response syndrome (SIRS), compensatory anti-inflammatory response syndrome, and mixed antagonist response syndrome. These reactions set in motion a cascade of complex processes involving also profound changes of the gene expression profiles (14). They are characterized by the release of cytokines and other inflammatory mediators and activation of cells of the adaptive immune system. NO and CO production is mediated by upregulation of nitric oxide synthase (NOS) and heme oxygenase (HO) predominantly *via* NF-κb (15) and *via* Nrf-2 pathways (16). Inflammatory mediators also affect tissue perfusion due to an interaction with the coagulation system (17) and effects on the vessel tonus, which may cause tissue hypoxia.

Aseptic inflammatory response occurs upon tissue damage that is induced either by mechanical trauma or hypoxia/ischemia reperfusion injury. Injured cells release danger signals (DAMPs), which subsequently activate an inflammatory response.

Upon trauma, both, PAMPs and DAMPs, initiate inflammation. Traumatic tissue injury results, on the one hand, in the loss of barrier function of the tissues, which opens the door for infections that set in motion septic inflammation mediated by PAMPs. On the other hand, tissue damage results in the loss of cellular integrity and the release of DAMPs, which induce aseptic inflammation. Inflammatory mediators induced by both, PAMPs and DAMPs, *via* interacting with specific membrane receptors, influence directly cellular pathways and are able to modulate mitochondrial function. However, inflammatory mediators, in particular NO, NO derived species, and CO also indirectly contribute to a further aggravation of the compromised tissue perfusion *via* their vasoactive effects (18). Thus, SIRS and tissue hypoxia can induce elevated NO and CO generation *via* several pathways.

**Figure 1:**
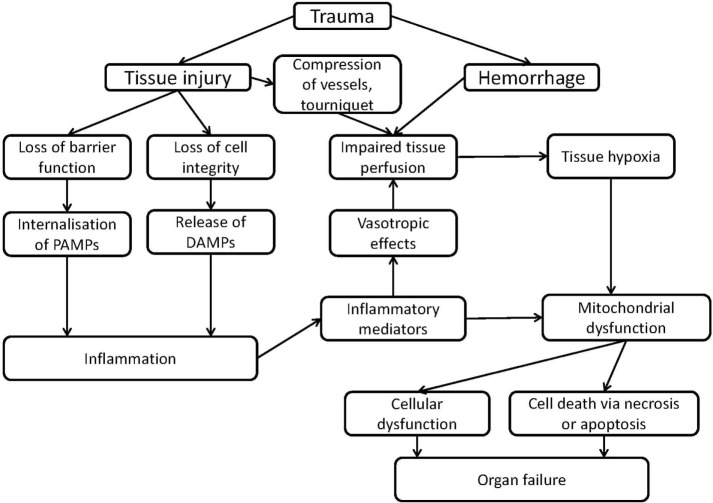
Scheme of mechanisms of organ failure induced by inflammation or trauma associated with mitochondrial dysfunction.

At a molecular level, two major hallmarks characterize the pathological sequelae in CCD: induction of tissue hypoxia and inflammation. Upon excessive hemolysis, iron toxicity may additionally occur. At cellular level, these factors dramatically influence the function of all subcellular structures. However, mitochondrial function is immediately and directly affected by the limitation of O_2_ and by the increased levels of NO and CO, which will further aggravate cellular and tissue dysfunction. The complex interaction of these processes, which may result in organ dysfunction is illustrated in Figure 1.

### Role of Hypoxia in CCD for NO and CO

Tissue hypoxia can be primary induced by mechanical pressure on blood vessels and reduced blood flow in tissues. It can also occur following posttraumatic hemorrhage and severe blood loss, which reduces blood volume and consequently compromises tissue perfusion. Under septic conditions, tissue hypoxia can be caused secondarily by inflammatory mediators, which induce vasodilation and tissue edema resulting in an insufficient tissue perfusion. Both, NO (19, 20) and CO (21, 22), cause vasodilation *via* interaction with soluble guanylate cyclase. In hypoxia, NO is generated primarily from nitrite (23, 24). Hypoxia also induces HO *via* hypoxia-inducible factor 1α pathway, and the subsequently increased HO activity leads to an enhanced CO generation at reoxygenation (25).

### Biological Activities of NO and CO

Both, NO and CO, are simple diatomic gases, which are endogenously produced in a variety of tissues throughout the body. In contrast to CO, which is chemically inert, NO is a free radical, and can react with a large number of molecules. In their gaseous form, NO and CO easily passage within cells and tissues by diffusion making them ideal signaling molecules. These properties result in a wide range of biological effects, of which the best known is the regulation of the vessel tonus exerted in the cardiovascular system. These signaling functions are described in detail in excellent recent reviews (22, 26–28) and will therefore not be discussed in detail in this review.

Both gases display a high affinity to iron and copper ions in metalloproteins, especially in hemoproteins (29, 30). In hemoproteins that require O_2_ for their enzymatic activities, the binding of NO or CO to the central iron ion in heme moieties results in an inhibition of the enzymatic activity due to the competition with O_2_. Displacing of O_2_ and inhibition of enzyme activity plays an important role for the modulation of mitochondrial function and may lead to enhanced generation of reactive oxygen species (ROS) and mitochondrial dysfunction (31, 32). While much is known about the mechanisms of NO- and CO-mediated regulation of mitochondrial enzyme activities under physiological conditions, there is much less information available about NO- and CO-mediated effects under conditions of CCD. Moreover, the effects of both gases can be both detrimental and beneficial, but the prerequisites required for these effects are still not clearly defined.

### Biosynthesis and Occurrence of NO and CO in Biological Systems

NO is synthesized by the three major isoforms of NO synthases (NOS), neuronal NOS (nNOS) endothelial NOS (eNOS), and inducible NOS (iNOS). Systemic inflammation is always accompanied by upregulation of iNOS, which is a prominent marker of inflammation (33). In contrast, constitutively expressed eNOS and nNOS can be activated by induction of Ca^++^ flow into the cell as a response to neuro- or inflammatory mediators or *via* parasympathetic nervous stimulation [reviewed in Ref. (34)]. Compounds containing transition metals are the major targets of NO in the body. Cytochrome c oxidase (COX) is the major target in mitochondria. There are two major complexes, namely NO–heme complexes and dinitrosyl complexes of free iron, which are formed upon binding of NO to such compounds. Nitrosyl complexes can be determined by electron paramagnetic resonance (EPR) spectroscopy. EPR spectroscopy is considered the most specific method to detect NO (35). The most sensitive is a chemiluminescence method, which is based on the reaction between NO and ozone. However, this method is less specific, as diverse compounds may emit chemiluminescence upon interaction with ozone as well. Another method is based on the electrochemical reduction of NO. The most used methods work with fluorescent dyes, such as DAF, which react upon interaction with NO. Such methods are generally easy to carry out, but they were shown to be less specific. Finally, NO levels can be determined in single cells by electrochemical methods using microelectrodes (36).

CO is produced by the inducible and constitutive isoform of heme oxygenase (HO-1, HO-2) from heme cleavage. In addition to CO, ferrous ions and biliverdin are released from this reaction. HO-1 is upregulated upon cell stressing conditions, and its expression is increased in CCD. Similarly to NO, iron-containing compounds, predominantly heme, are the major targets of CO in the body. COX is the major target in mitochondria. CO is chemically an inert gas, and endogenously produced CO does not accumulate, but diffuses into the blood stream and forms carboxy-hemoglobin (CO-Hb). CO competes with O_2_ for the same binding sites, but displays a several hundred-fold higher affinity to Hb. Due to its characteristic absorption properties, CO can easily be determined in the blood by photospectroscopy. CO is liberated *via* the lungs where O_2_ levels are sufficiently high and leaves the body with the exhaled air. As a gas, CO can be determined by gas chromatography with reductive (mercuric oxide) detection. Both, determination of CO-Hb in blood and CO in the exhaled air (37) can give valuable information about the endogenously generated amount of CO under standardized conditions (38). Frequently, the determination of the enzyme activities are used to get information about the capacity to produce NO or CO levels in tissues or cells. However, these biochemical assays are always carried out in an access of substrates and co-substrates, and the generated levels of CO or NO do not necessarily reflect the situation *in vivo*. However, a compromised enzyme function or an enhanced capacity to generate products may be determined in this way.

### The Concentrations of NO and CO Determined in CCD (Sepsis, Hemorrhagic Shock, and TBI)

In the blood of septic patients, the concentrations of NO were 40–80 µM, while those of CO were reported to be in the range of 0.035–0.175 µM (39). Mean plasma levels of NO derivatives, a sum of nitrite and nitrate concentrations, in septic patients reach 70–150 µM (40), while in rodents, NO levels can be much higher reaching concentrations of several millimolar (41). Upon HTS, the concentration of NO can also be elevated but substantially less compared to sepsis. In a rodent model of TBI, the concentration of NO determined in cortex was about 4 µM (42).

It has been shown that under physiologic conditions, humans produce 0.4 ml/h (18 µmol/h) CO, which increases to 3.6 ml/h (143 µmol/h) under pathophysiologic circumstances (43). Exhaled CO is used as a biomarker of inflammation in CCD or to monitor the effectiveness of pharmaceutical interventions [for review, see Ref. (44)]. Enhanced exhaled CO levels were shown in critical care patients under inflammatory conditions (45). Especially during the initial course of sepsis endogenous CO production was increased. These levels correlated with the increased levels of CO-Hb and bilirubin in plasma (46, 47), indicating an increased heme breakdown by HO in the septic patients. Interestingly, in this study, survivors of sepsis displayed an initially higher endogenous CO production compared to non-survivors (38, 48). Another study investigating the continuously monitored CO-Hb values in the blood of critically ill patients confirmed this association (49). These findings point towards a beneficial role of an enhanced HO reaction in the very initial phase of systemic inflammation (46, 48). This aspect suggests application of CO as a possible therapeutic approach to treat pathologies associated with underlying inflammatory complications [for review, see Ref. (50)].

Thus, the concentrations of NO and CO occurring in CCD are at micromolar range. However, several other factors, first of all the concentration of O_2_, determines whether such concentrations are sufficient to affect mitochondrial function.

The interaction of NO and CO with biomolecules including COX is reversible, which is the premise for acting as intracellular regulatory signaling molecule. In CCD, not only NO and CO production is enhanced but also simultaneously O_2_ tension is decreased. Thereby, the equilibrium between O_2_ and gaseous messengers is shifted toward the gaseous messengers, which amplifies the NO- and CO-mediated effects. Thus, the hypoxic compartments in CCD patients are particularly threatened to encounter critical concentrations that result in unfavorable effects of the gaseous messengers.

### Tissue Oxygenation in CCD

In normoxic organs, the concentrations of O_2_ are approx. 30 µM (20 mmHg), which is much less than those reached under ambient conditions (about 250 µM, 160 mmHg). The levels of intracellular O_2_ concentration vary and are classified in accordance to their physiological actions as follows (24). Hyperoxia is defined as [O_2_] >130 μM, normoxia is the interval between 130 and 20 µM of O_2_. Oxygen deficiency (hypoxia) is defined as the interval of O_2_ levels between 20 and 2 µM. At these O_2_ concentrations, mitochondria still are able to synthetize ATP. The range of 0–2 µM of O_2_ is defined as anoxia. At these concentrations, the mitochondrial respiration is severly compromised, and consequently, the intracellular metabolism is profoundly affected.

It has been shown that both septic and hemorrhagic shock cause a decrease in tissue O_2_ levels. Using fibreoptic O_2_ micro-sensor, Dyson and coauthors compared the O_2_ tension in different organs of rats subjected to endotoxemia and controlled hemorrhage. Upon septic shock, the levels of O_2_ in muscle, bladder, liver, and kidney were in average two times lower than those in the control animals (51). The most pronounced changes were observed in liver. Upon hemorrhage the O_2_ levels in muscle and liver decreased more then 10-fold, while in bladder and kidney the decrement was less pronounced (51).

### Hypoxia Enhances Effects of Gaseous Messengers on Mitochondria

It has been shown that intestinal mucosal hypoxia (PO_2_ < 20 mmHg) occurs in hemorrhagic shock already at a mean arterial pressure <60 mmHg (52). In another study, lipopolysaccharide (LPS) infusion in pigs was shown to decrease mucosal tissue O_2_ tension from a PO_2_ of 24 ± 3 to 12 ± 2 mmHg (53). A similar effect, a drop in O_2_ levels from 28 mmHg to approx. 15 mm Hg was observed in the cortex of rats subjected to a moderate controlled cortical impact (54). Thus, all CCD result in decreased tissue O_2_ levels, which are more pronounced in hemorrhagic shock. This observation leads to the assumption, that production of NO and CO is decreased in hypoxia, since NOS and HO both require O_2_ to synthesize NO and CO, respectively. However, the lower rate of synthesis will probably be compensated by a higher efficiency of the gas messengers to compete with the remaining O_2_ for the binding at COX. Additionally, the low levels of synthesis of these gas messengers may be compensated by the release of NO and CO from other sources. CO and NO diffuse out of the cell into the circulation and are trapped by Hb. Hb serves as a sink or a buffer for NO and CO and constitutes a pool of bound gaseous messenger (55). However, this pool of bound NO/CO can become a source of NO under hypoxic conditions. In hypoxic environment NO is released from Hb (from S-NO (56)); this process is facilitated by protein disulfide isomerase (57). Released NO exerts vasotropic effects, which alter tissue perfusion. Alternatively under anoxic conditions, NO can be formed from nitrite (24, 58) further increasing NO levels. The combination of these factors determines the strength of the modulatory effects on mitochondrial function by NO and CO themselves. Additionally, NO can impair mitochondrial function *via* secondary products formed predominantly in reactions of NO with ROS.

### ROS Generation in CCD

There are several sources of ROS in the cell. The major sources are mitochondria, NADPH oxidase, cytochrome P450, peroxy-somes, and xanthine oxidase. Mitochondria are considered as the main source of ROS in the majority of parenchymal cells, while NADPH oxidase is the most powerful generator of ROS in cells of the innate immune system. Although ROS derived from NADPH oxidase contributes to the intracellular ROS pool to a certain degree, the largest portion of ROS is released into the extracellular space. Elevated ROS generation was described in sepsis (59) and HTS (60) as well as in TBI [reviewed in Ref. (34)]. ROS generation occurs at the late phase of HTS, during resuscitation and reoxygenation and after the activation of immune cells upon inflammation. Also in TBI, the generation of reactive oxygen and nitrogen species (RONS) is elevated (34, 61). ROS has been associated with a negative outcome in TBI (62), HTS (63, 64), and sepsis (65). NO, in contrast to CO, reacts with ROS. The interaction between NO and superoxide radicals, the primary ROS generated by mitochondria, results in the formation of peroxynitrite (ONOO), a secondary ROS which is toxic, causing irreversible oxidative damage to biomolecules [for review, see Ref. (66)]. Mitochondrial proteins predominantly affected are those at complexes I and II of the electron transport chain (67). Such damage can facilitate the opening of the mitochondrial permeability transition pore, which in turn disrupts ATP synthesis and induces apoptosis (18, 68). Thus, excessive formation of ROS in CCD will favor deleterious effects of NO.

### Tissue Acidosis in CCD and Its Effect on NO and CO

Acid/base and electrolyte disorders are very common in CCD and constitute an additional risk factor in these patients, particularly if metabolic acidosis develops (69, 70). A retrospective study revealed 59% mortality in a group of CCD patients with metabolic acidosis compared to patients with non-lactic acidosis (71). The severity of lactic acidosis in critically ill patients correlates with overall O_2_ debt and inversely with survival (70). Lower pH favors the release of O_2_ from Hb and will favor the formation of nitric acid from nitrite. It has been shown that nitric acid can easily diffuse through biological membranes and become an additional source of intracellular NO in hypoxic conditions (24). The formation of NO from nitrite occurring in hypoxic/ischemic part of the body slows down mitochondrial respiration (72) but simultaneously protects mitochondria by inhibiting iron-mediated oxidative stress occurring under hypoxic conditions (23). As outlined before, CO and NO, due to their high affinity, are scavenged by Hb and myoglobin under physiological conditions. These molecules therefore provide an effective protection against the possible inhibition of cellular respiration by free CO and NO (73). However, stability of the heme protein complex with CO (74) and NO (24) display a strong pH dependency, similarly to O_2_. Acidic pH favors the O_2_/CO partition toward O_2_, which means that the formation of CO-Hb requires higher CO concentration (75). This suggests that tissue acidosis may reduce the flow of CO into the blood to form CO-Hb facilitating reactions of CO with intracellular heme proteins, including COX (76), with a subsequent inhibition of mitochondrial respiration (73).

### Mitochondrial Function/Dysfunction in CCD Models

Most findings regarding changes of mitochondrial function were obtained using experimental models for CCD, frequently in rodents. Sepsis has been induced by injection LPS or by induction of peritonitis, generally *via* cecal ligation and puncture (CLP) and instillation of feces in the abdominal cavity [for review, see Ref. (77, 77, 78)]. Data regarding mitochondrial function using these models are inconsistent. While some studies show unchanged or even elevated respiration rates (79–82), others reported a decrease in respiration rate (11, 83, 84). In porcine models, which are considered better suited to mimic the pathophysiological situation in humans, mitochondrial dysfunction was reported more consistently, although the differences determined were minor compared to those found in some rodent models. Changes were determined mostly in liver, heart, and brain (85–87). The relevance of mitochondrial dysfunction determined in experimental animal models in the view of the reported conflicting results has recently been addressed in a review by Jeger and coauthors (13). However, some of the differences determined have to be attributed to the method applied for measuring mitochondrial dysfunction.

### Methods to Detect Mitochondrial Function/Dysfunction

The determination of mitochondrial function in tissues can be approached in two different ways. While the determination of ATP levels gives an idea about the activity of mitochondria at the respective time point and inside the tissue, it is an indirect measure for the underlying mitochondrial function. No information is obtained whether and at which site a possible functional defect may be present. Examination of mitochondrial function *ex vivo* is achieved by measuring O_2_ consumption in presence of saturating concentrations of the respective substrates and in combination with complex-specific inhibitors (88, 89). This analysis allows the determination of the activities of different enzymes of the mitochondrial electron transport chain and TCA cycle (90, 91). It is therefore a measure for the capacity of mitochondrial function in a given tissue. As discussed above, mitochondrial dysfunction can be the result of a reversible or/and irreversible inhibition. Decreased levels of ATP in tissues determined *in vivo* will be measured irrespectively whether inhibition of mitochondrial function was reversible or irreversible. Using *ex vivo* approaches, i.e., analyzing homogenated tissues or isolated mitochondria, mitochondrial dysfunction will only be determined, if the inhibition was irreversible.

For instance, decreased levels of tissue ATP in combination with an unchanged respiratory function of mitochondria determined *ex vivo* were reported in a rodent model of endotoxic shock (92). These data indicate that mitochondrial function was reversibly inhibited at the investigated time point. Reports on the determined ATP levels in tissues upon systemic inflammation are much more consistent, compared with those investigating mitochondrial respiration. They show an approximately twofold decrease (18). This fact is satisfactorily explained by reversible inhibition of mitochondrial function in the tissue by NO and/or CO.

As outlined above, both CO and NO have high affinity to the COX at the O_2_-binding sites and will replace O_2_ in the tissues at low O_2_ levels. During isolation of mitochondria or during preparation of tissue homogenates mitochondria are exposed to an ambient O_2_ concentration, resulting in oxygenation of COX. Thus, CO/NO-mediated inhibition of mitochondria, which is reversible, will not be determined in isolated mitochondria. By contrast, an irreversible inhibition will result in both, decreased ATP levels and rates of mitochondrial respiration determined the *ex vivo*. Interestingly, irreversible inhibition of mitochondria has been reported more consistently in porcine and feline sepsis models, compared to experimental models in rodents. In pigs, the endogenous levels of NO are physiologically lower suggesting that even low ROS levels can turn the entire NO pool into ONOO causing irreversible damage to mitochondria. That can explain occurrence of irreversible impairment of mitochondrial respiration in those species.

### Possible Mitochondria Mediated Beneficial Effects of Gaseous Messengers on the Outcome in CCD

The interaction between NO/CO and mitochondria may not only be deleterious but also beneficial. It has been shown that mitochondrial biogenesis can be stimulated by NO (93) and CO (94). This can facilitate the recovery of mitochondrial function. Both CO and NO by competing with O_2_ temporarily inhibit COX and thereby inhibit ATP production. Interestingly, an approximately twofold decrease in ATP levels can prevent apoptosis. This effect may be beneficial and explains the observation of an induced apoptosis, which was not executed in a model of endotoxic shock (95).

The CO/NO triggered generation of mitochondrial ROS appears to have also an important beneficial role. A recent study shows that application of mitochondria-targeted ROS scavengers may be harmful in a CLP model (96), although they proved beneficial in a model of endotoxic shock (97). It has been shown that NO can stimulate ROS production in a feed forward manner where ROS upregulate iNOS. This effect results in elevated generation of NO, which in turn elevate mitochondrial ROS generation (97). A similar feed forward mechanism involving HO-1 was hypothesized for CO (98). RONS formed from NO (if intracellular redox potential stimulates excessive ROS generation) can uncouple mitochondria and induce mitochondrial permeability transition pore opening. However, mitochondria can also recycle NO from nitrite facilitating tissue perfusion and protecting tissue against oxidative stress (99).

**Figure 2:**
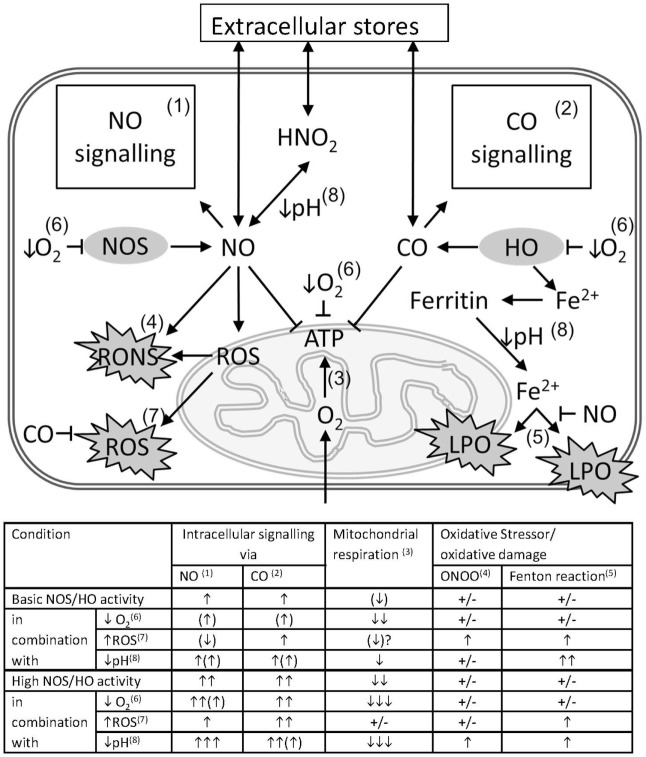
Pathological impact of the interference of gaseous messengers NO and CO with mitochondria in critical care diseases (CCD). The schema illustrates the possible interactions between the gaseous mediators NO and CO with mitochondria in conjunction with CCD associated factors that exert a strong interfering potential. Intracellular concentrations of NO and CO are determined by the underlying enzyme activity of NOS and HO and by diffusion from or to extracellular stores. NO and CO mediate intracellular signaling pathways (1 and 2), such as activation of guanylate cyclase, ion channels, and activation of gene signatures, etc. Both messengers are able to inhibit mitochondrial respiration (3), mainly by competing with O_2_ for binding to cytochrome c oxidase (COX). NO in conjunction with reactive oxygen species (ROS) is able to from peroxynitrite (ONOO) (4), with the capacity to damage biomolecules. Free ferrous ion is able to induce lipid peroxidation (LPO) *via* the Fenton reaction (5) in presence of ROS. By the formation of nitrosyl species, NO may prevent iron-mediated oxidative damage. Scarcity of O_2_ resulting from tissue hypoxia (↓O_2_), decelerates all oxygen-dependent reactions (6), and leads to a decreased formation of ATP. In hypoxia also the intracellular production of NO and CO is diminished, and the intracellular levels of NO and CO are supposed to decrease as well. However, the high consumption of oxygen will overrule this effect and presumably shift the balance towards NO- and CO-mediated inhibition of mitochondrial respiration. Intracellular ROS may originate from dysfunctional mitochondria, especially in presence of increased levels of NO, or from non-mitochondrial sources, and exert pro-oxidative damaging effects (7). These effects may be limited by the HO reaction products CO and biliverdin/bilirubin. Acidosis (↓pH) (8), which may occur secondary to tissue hypoxia, favors the formation of nitric acid from NO oxidation products, such as nitrite or nitrate, allowing diffusion across membranes. Additionally, a decreased pH affects the affinity constants of biatomic gases to the heme moiety to different degree. For hemoglobin, which is considered as a sink for CO, a decreased CO affinity will result in increased levels intracellular CO and an increased potential to inhibit mitochondrial respiration. The table attempts to quantify the effects of CCD-associated factors, such as tissue hypoxia, increased generation of ROS (↑ROS), and tissue acidosis on the interactions between the gaseous mediators NO and CO with mitochondria. Superscripted numbers refer to the interaction or pathway shown in the scheme. The number of arrows represents the presumed amplitude of the indicated effect. NO and CO influence on intracellular signaling, mitochondrial respiration, the generation and the effects of oxidative stressors, such as ONOO and ROS, involving the Fenton reaction, to different degree.

Summarizing the data described in this review, we assume that the result of interaction between gaseous mediators and mitochondria, which is visualized in Figure 2, is predominantly defined by the following four conditions.

(1) The enzymatic systems generating gaseous messengers are often dysregulated under hypoxia and inflammation. If produced in excess, they compete for the O_2_-binding sites and may substantially impair mitochondrial respiration.
(2) O_2_ saturation of tissues and mitochondrial access to O_2_ is frequently limited in CCD. Thus, the effects of CO and NO are more pronounced at low O_2_ concentrations. *Vice versa*, increased O_2_ levels will weaken the effects of the gaseous messengers. Apart from mitochondrial proteins, gaseous messengers can also directly influence gene expression *via* reaction with heme-containing transcription factors and thereby activate mitochondrial biogenesis.
(3) Redox state of the cell and mitochondria is frequently severely deranged. Changes in the redox environment of the cell are able to influence the interaction of gaseous messengers with mitochondria. CO is not redox active, and its effects are not likely influenced by redox status. By contrast, NO is redox active and can generate further products, which have quite different chemical properties than NO itself. NO reacts with superoxide to form ONOO, which is a strong pro-oxidative molecule inducing oxidative stress, and irreversibly damages complex I, aconitase, and a number of other mitochondrial proteins. It can also damage mitochondrial membrane and uncouple mitochondria. By contrast, NO itself exerts antioxidative properties by binding free ferrous iron and preventing mitochondrial damage *via* the Fenton reaction (23). Therefore, the reaction of NO strongly depends on the redox potential of the cell. The balance between the effects of NO or ONOO will be shifted favoring those of ONOO, if the intracellular redox potential favors formation of ROS.
(4) Frequently a dysregulation of the acid/base balance toward acidosis is determined in CCD. Hypoxia/ischemia leads to an enhanced anaerobic glycolysis due to the scarcity of oxygen. The resulting increased production of lactate is associated with a decrease in tissue pH. Lower pH favors the formation of nitric acid from nitrite, the end product of the NO catabolism. Under acidic conditions, nitric acid can disproportionate and thereby release NO (100). Therefore, NO concentration is higher in acidic hypoxic tissues (100).

## Conclusion

Thus, the mitochondria mediated actions of NO and CO can be both deleterious and beneficial. There are four major factors determining their efficiency, concentration of mediators, O_2_ levels, redox status of cells facilitating or inhibiting ROS formation, and acidosis. High levels of gaseous messengers and a redox status favoring ROS formation shift the balance in favor of deleterious reactions. This occurs particularly in ongoing inflammatory reactions. By contrast, hypoxic/anoxic conditions possibly favor NO/CO-mediated beneficial effects. However, upon reoxygenation during reperfusion, which is associated with enhanced ROS release, the deleterious effects of NO may prevail due to the formation of RONS. The effects of CO on the other hand are expected to be beneficial under these conditions. Thus, the effects of NO/CO are dependent on the balance between the NO/CO generation and elimination rates, ongoing inflammatory responses, and the generated ROS as well as the depth of tissue hypoxia.

## Author Contributions

JCD contributed by writing predominantly on CO and heme oxygenase, general editing and approval of the manuscript. AVK contributed by writing predominantly on NO and nitric oxide synthase, general editing and approval of the manuscript.

## Conflict of Interest Statement

The authors declare that the research was conducted in the absence of any commercial or financial relationships that could be construed as a potential conflict of interest.
